# Friendship jealousy and interaction needs: how mutual friend features affect users of WeChat Moments

**DOI:** 10.3389/fpsyg.2024.1411034

**Published:** 2024-10-09

**Authors:** Yehan Zou, Xiqing Han, Xudan Yu, Yun Zhang, Qinghong Shuai

**Affiliations:** ^1^School of Business Administration, Southwestern University of Finance and Economics, Chengdu, China; ^2^School of Information Management, Wuhan University, Wuhan, China; ^3^School of Management Science and Engineering, Southwestern University of Finance and Economics, Chengdu, China; ^4^School of Management, Tianjin University, Tianjin, China; ^5^Engineering Research Center of Intelligent Finance, Ministry of Education, Southwestern University of Finance and Economics, Chengdu, China

**Keywords:** mutual friends, friendship jealousy, interaction needs, social networking services, information dissemination

## Abstract

Many social networking services (SNSs) have features that highlight the common friends of pairs of users. Previous research has examined recommendation systems that use mutual friend metrics, but few scholars have studied how the existence of features related to mutual friends affects users in SNSs. To explore this issue further, we conducted interviews with 22 users of WeChat Moments to investigate how certain rules involving mutual friends affect users and how they deal with the issues that arise due to these rules. We found that the three Moments rules related to mutual friends (response visibility, response notifications, and information dissemination) can cause users to feel jealous, annoyed, and embarrassed. To prevent these negative experiences, users may reduce the amount of information they disclose or the frequency of their interactions in SNSs. Based on these findings, we propose several future directions for scholars and a small number of design suggestions aimed at assisting providers to satisfy users’ interaction needs.

## 1 Introduction

Social networking services (SNSs) break space limitations by mapping different circles of friends onto a mostly flat structure. This is known as “context collapse” (Marwick and Boyd, 2011). In this context, many pairs of SNS users find that they have mutual friends. Knowing about these mutual friends may improve the level of trust between two users, especially when the relationship between the users is relatively distant (Nagy et al., 2013). Because of this, most SNS providers use a “mutual friends” metric to develop their recommender systems (Chen et al., 2009). For instance, the feature “People You May Know” on Facebook recommends new friends for users based on the “friend-of-a-friend” rule. Previous research on mutual friends has mainly focused on the friend recommendation algorithm, exploring how users make interesting new friends through mutual friends (Chen et al., 2009; Nagy et al., 2013; Chiou and Huang, 2013; Fu et al., 2014). Some scholars have developed specific tools (e.g., “Common Friend-Finder”) to estimate the number of common friends between pairs of users (Fu et al., 2014). Others have explored people recommender systems in SNSs using a range of algorithms including collaborative filtering (which is based on the similarities of preferences among users (Herlocker et al., 2000)) and articulated social network structures (Groh and Ehmig, 2007). It is important to examine whether the implementation of mutual friend features has the desired results.

WeChat Moments (hereafter referred to as “Moments”) is the most popular social media platform in China (Zhang et al., 2024). Similar to Facebook’s Timeline, Moments allows users to share status updates, photos, and videos. However, Moments is a semi-closed platform where only individuals in a user’s contact list can view the posts, making it a more private way to share content with friends and family. Specifically, Moments has three features associated with common connections. First, members of a particular user’s audience can only view responses to posts that are made by mutual friends. That is, a user cannot see all of the feedback on a certain post on Moments—they can only see comments from people who are friends with both the user and the poster. Second, when a member of the audience comments on a post, he or she receives notifications about subsequent responses from other mutual friends. Third, like most social media, there are no settings on Moments that prevent contact between mutual friends.

Mutual friends may influence users’ perceptions and behaviors in SNSs (Green et al., 2018; Zhang et al., 2021a), however few studies have explored the rules related to mutual friends in depth. In the present study, we aimed to gain a deeper understanding of the issues caused by mutual friend rules in SNSs. We structured our study around the following research questions:

RQ1: How do the mutual friend rules on Moments affect users?

RQ2: How do users deal with mutual friend issues on Moments?

We found that the rules on Moments related to mutual friends may lead to negative experiences for users. First, response visibility (i.e., the fact that users can only see responses from mutual friends) may lead users to make relationship comparisons. For instance, poster A might compare the likes and comments received from mutual friends with poster B and feel jealous or disappointed if there is lower engagement. Second, users may be annoyed by notifications based on the activity of mutual friends. The participants in our study commented, for example, that they did not want to receive low-effort responses (i.e., likes) from mutual friends. The mutual friend rule may also lead to the dissemination of information to an unintended audience, which can expose whether the poster has blocked someone and lead to potentially embarrassing situations.

We make the following contributions to the social media literature. First, as mentioned earlier, mutual friend features may affect users’ attitudes and behaviors when using SNSs, but few studies to date have investigated this issue in detail. People are collecting an increasing number of online friends, and this increase may exacerbate any negative outcomes. We therefore extend existing studies on the “dark side” of social media and shed new light on mutual friend issues in SNSs. Our results also serve as a call to providers to design more helpful algorithms and more effective features that address users’ concerns about the activity of mutual friends in their social networks.

## 2 Related work

### 2.1 Prior research on mutual friends in SNSs

Previous research on mutual friends has mainly focused on the friend recommendation algorithm, exploring how users make interesting new friends through mutual friends (Chen et al., 2009; Chiou and Huang, 2013; Fu et al., 2014; Nagy et al., 2013). For instance, the “People You May Know” feature on Facebook recommends potential new friends to users based on the “friend-of-a-friend” algorithm. Some scholars have developed specific tools (e.g., “Common Friend-Finder”) to estimate the number of common friends between pairs of users (Fu et al., 2014). Others have explored people recommender systems in SNSs using a range of algorithms including collaborative filtering (which is based on the similarities of preferences among users) and articulated social network structures (Groh and Ehmig, 2007; Herlocker et al., 2000). For instance, Tinder recommends potential partners to users based on geospatial proximity and “common connections” (i.e., shared Facebook friends) (Green et al., 2018).

However, only a small number of studies have focused on the effects of mutual friend rules on social media use (Green et al., 2018; Li et al., 2018). On the one hand, knowledge about mutual friends could facilitate interactions between strangers. For instance, Green et al. (2018) claimed that the “common connections” feature on Tinder affected users’ sexual decision-making processes. Specifically, users may see partners with whom they have mutual friends as safer and more familiar, which may lead to increased sexual risk-taking behaviors. On the other hand, as users’ audience sizes increase, features based on the mutual friends of pairs of users may lead to negative outcomes. For example, Li et al. (2018) found that mutual friend features increase the burden of audience segmentation and may lead users to apply the Time Limit setting, which automatically hides content posted on Moments after a short time. Zhang et al. (2021a) found that many users were concerned that people they had blocked could still learn about their posts through interacting with mutual friends via other channels. These conflicting findings suggest that more research is needed on the effects of mutual friend rules on social media platforms.

### 2.2 SNS use and jealousy

Jealousy is a “complex of thoughts, feelings, and actions which follow threats to self-esteem and threats to the existence or quality of a relationship” (White, 1981). Jealousy can be viewed as being comprised of several emotions, including anger, sadness, anxiety, and embarrassment (Parker et al., 2005; Parrott and Smith, 1993). Prior research has indicated that SNS use triggers jealousy, especially in romantic relationships (Daspe et al., 2018; Muise et al., 2009; Utz and Beukeboom, 2011). This is believed to be because spending more time on SNSs increases the chance that someone will encounter ambiguous information that can be misinterpreted and evoke jealousy (Bevan, 2013; Hudson et al., 2015).

Studies have mainly focused on romantic jealousy in SNSs, exploring its potential antecedents and consequences (Demirtaş-Madran, 2018; Hudson et al., 2015; Marshall et al., 2013; Orosz et al., 2015). For instance, Elphinston and Noller (2011) found that Facebook intrusion increased romantic jealousy. Social grooming behaviors (e.g., leaving comments on a post) may also increase SNS-related jealousy (Utz and Beukeboom, 2011). Marshall et al. (2013) found that individuals with high levels of attachment anxiety experienced more Facebook jealousy due to their difficulties in trusting others. Other scholars have argued that individuals with low self-esteem and a high need for popularity are more likely to feel jealous (Demirtaş-Madran, 2018; Utz and Beukeboom, 2011). Jealousy can cause negative relationship outcomes, such as relational aggression (Murphy and Russell, 2018), relational conflicts (Fox et al., 2013) and intimate partner violence (Daspe et al., 2018). It can also lead to surveillance behaviors on Facebook (Marshall et al., 2013). It has been argued that females are more likely than males to have jealousy-related feelings and behaviors triggered by SNSs (McAndrew and Shah, 2013; Muise et al., 2009; Muscanell et al., 2013).

Some scholars have claimed that individuals also experience jealousy related to friendships (i.e., friend jealousy (Aune and Comstock, 1991; Bevan, 2013)). Studies have shown, for example, that girls and adolescents with low self-worth experience more friendship jealousy (Parker et al., 2005). Jealousy in friendships has been found to be associated with conflicts, disconnectedness, and aggression, and it is argued to damage the quality of friendships and reduce prosocial behavior toward friends (Deutz et al., 2015; Kraft and Mayeux, 2018; Lavallee and Parker, 2009). Other scholars have argued that friendship jealousy can be used as a tool for maintaining friendships, as it allows people to hold onto friends for longer (Krems et al., 2021). Although several studies have found that SNS use may lead to jealousy, these studies focused only on romantic jealousy. However, given that jealousy also exists in friendships, we can predict that certain information may give rise to friendship jealousy.

## 3 Mutual friend rules in SNSs

This paper primarily examines the impact of mutual friend rules on users, using WeChat as an example. Therefore, we will elaborate on the mutual friend rules specific to the WeChat platform. Additionally, to enhance the universality of our findings, we have also compared the mutual friend rules of WeChat with those of other popular social media platforms (i.e., Facebook and Instagram).

### 3.1 Mutual friend rules on WeChat Moments

WeChat Moments enables users to create a profile, post content, and interact with others. To improve user activity, Moments has introduced three rules that are directly related to mutual friends: response visibility, response notifications, and information dissemination.

#### 3.1.1 Response visibility based on mutual friends

**FIGURE 1:**
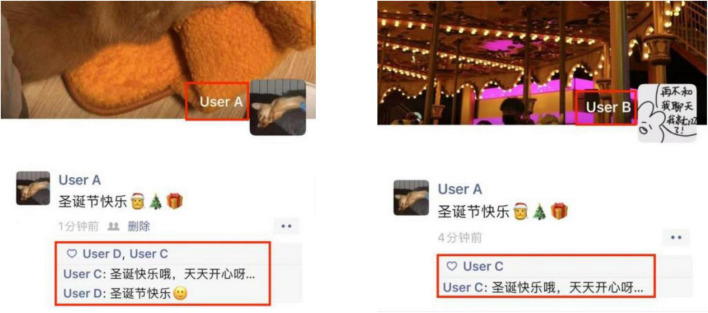
Response visibility based on mutual friends.

The first rule relating to mutual friends is response visibility. Moments is a semi-closed sphere in which responses (i.e., likes and comments) on a post can only be viewed by mutual friends. In other words, audience members do not see all of the feedback on a post. For instance, consider the situation in which Users A, B, and C are mutual friends on Moments (i.e., Users A and B, Users A and C, and Users B and C are WeChat friends). When User A shares a post, User B can only see responses from mutual friends (e.g., User C) and cannot view other users’ responses (e.g., those of User D, as shown in Figure 1).

#### 3.1.2 Response notifications based on mutual friends

**FIGURE 2:**
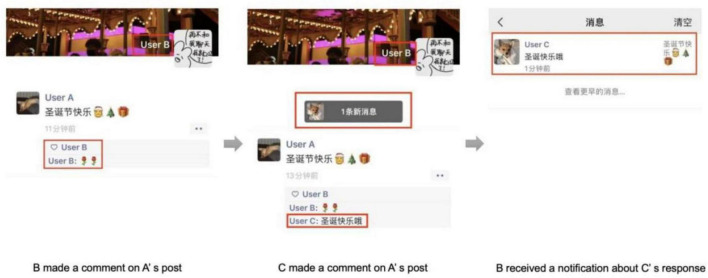
Response notifications based on mutual friends.

The second rule related to mutual friends consists of response notifications. Specifically, when a user leaves a response to a post, he or she receives notifications when there are subsequent responses from other mutual friends. For example, consider again the situation in which Users A, B, and C are mutual friends. If User C leaves a comment on User A’s post after User B has commented, User B receives a notification about User C’s response (as shown in Figure 2).

#### 3.1.3 Information dissemination based on mutual friends

**FIGURE 3:**
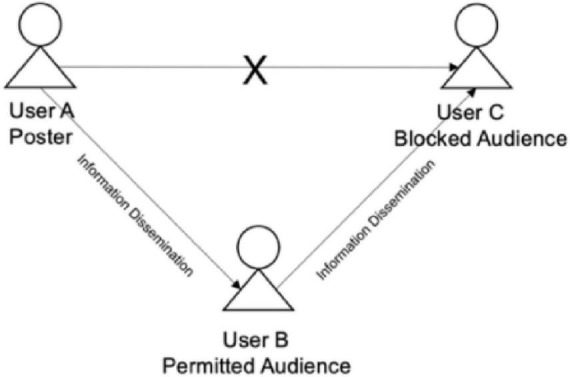
Information dissemination based on mutual friends.

The third rule related to mutual friends is linked to information dissemination. Specifically, most social media platforms do not provide relevant norms or features by which users can prevent the dissemination of information between mutual friends. That is, the content of a user’s post might be shared with blocked connections by a friend who has not been blocked. For instance, consider again the situation in which Users A, B, and C are mutual friends. User A shares a post with a selected audience only. User B (part of the permitted audience) can view this post, but User C (part of the blocked audience) has no access to it. However, User B may talk about User A’s post with User C, which exposes the fact that User C has been blocked by User A (as shown in Figure 3).

### 3.2 Comparison of interaction rules across social media platforms

In the realm of social media, interaction rules significantly influence user engagement. These rules vary noticeably across different platforms, reflecting their unique strategies in fostering user interactions. To enhance the general applicability of our research findings, we specifically compare the interaction rules related to mutual friends of WeChat with those of two other mainstream social platforms—Facebook and Instagram.

As shown in Table 1, it can be observed that WeChat Moments, being a semi-closed social platform, tends to be more private in its interaction rules compared to Facebook and Instagram. Specifically, Moments’ response visibility and response notifications are designed based on mutual friends, while the other two platforms default to settings based on all friends. Additionally, none of the three major platforms provide features or rules to prevent the dissemination of information among mutual friends. Moreover, the mutual friend rules of Facebook and Instagram primarily manifest in displaying the number of mutual friends between users, a feature not available in Moments. Overall, while Facebook and Instagram do not specifically tailor response visibility and response notifications based on mutual friends, their settings based on all friends encompass mutual friends as well. Therefore, issues related to mutual friends are also likely to arise on these social platforms.

**TABLE 1:** Comparison of interaction rules across social media platforms.

Rules	WeChat Moments	Facebook	Instagram
Response visibility	Visible to only mutual friends.	Visible to all friends.	Visible to all friends.
Response notifications	Receives notifications for subsequent responses from only mutual friends.	Receives notifications for all subsequent responses.	Receives notifications for all subsequent responses.
Information dissemination	Unable to prevent information dissemination among mutual friends.	Unable to prevent information dissemination among mutual friends.	Unable to prevent information dissemination among mutual friends.
Mutual friends count	Don’t display the number of mutual friends between users.	Display the number of mutual friends between users.	Display the number of mutual friends between users.

## 4 Methods

To gain a deep understanding of how mutual friend rules affect users on Moments, we conducted a series of semi-structured interviews. We initially recruited participants through personal contacts and then contacted more using a snowballing technique. The interviews were conducted in Mandarin Chinese, the mother tongue of both the interviewers and interviewees. Each interview began with an introduction to the mutual friend rules on Moments. We then asked the participants about their general use of Moments and their opinions about response visibility, response notifications, and information dissemination based on mutual friends. We asked questions to determine their opinions as both posters and audience members (e.g., “How do you feel when you see a mutual friend respond to others’ posts but not to yours?”) and how they dealt with the issues caused by these rules (e.g., “How do you deal with the possibility that a friend may share the content of your post with a mutual connection who you have blocked?”). The general interview questions are provided in the Appendix A. We also gathered basic information about the participants (gender, age, occupation, and frequency of Moments use), which is shown in Table 2.

**TABLE 2:** Basic participant information.

Name	Gender	Age	Occupation	Average time per day they used Moments	Frequency of Moments posts	Number of WeChat contacts
P1	F	21	Student	2 h	Weekly	100
P2	M	22	Student	4 h	Weekly	759
P3	F	23	Piano teacher	2 h	Monthly	720
P4	F	22	Student	2 h	Daily	340
P5	F	20	Student	1 h	Yearly	200
P6	M	21	Student	4 h	Monthly	280
P7	F	21	Student	3 h	Monthly	311
P8	F	48	Clerk	3 h	Weekly	348
P9	M	50	Clerk	3 h	Daily	573
P10	F	21	Student	4 h	Weekly	125
P11	F	22	Student	2 h	Daily	207
P12	F	21	Student	1 h	Weekly	556
P13	M	22	Student	2 h	Weekly	421
P14	M	21	Student	3 h	Weekly	295
P15	F	20	Student	2 h	Weekly	126
P16	M	21	Student	2 h	Monthly	231
P17	F	21	Student	1 h	Weekly	786
P18	F	20	Student	3 h	Weekly	201
P19	F	20	Student	2 h	Daily	311
P20	F	20	Student	3 h	Weekly	432
P21	F	21	Student	1 h	Monthly	220
P22	F	22	Student	0.5 h	Weekly	556

We conducted 22 semi-structured interviews lasting between 20 and 40 min (M = 35). We stopped conducting interviews upon reaching theoretical saturation. The participants received RMB 20 as a token of our appreciation. In total, there were 16 female and 6 male participants, ranging in age from 19 to 50. Most of the participants were university students, a demographic that is more likely to contain active social media users (Huang et al., 2020). All of the interviews were audio-recorded. They were then transcribed using the software package Iflyrec, with two researchers listening to the recordings sentence by sentence to verify the accuracy of the transcription and make note of important information.

Adopting the thematic analysis method as outlined by Braun and Clarke (2006), our analysis process unfolded systematically. First, two researchers engaged in multiple rounds of open coding, meticulously sifting through the data. This initial phase involved deep immersion into the audio recordings and transcripts, enabling the identification of key concepts directly relevant to our research questions. Second, these insights laid the groundwork for the identification and refinement of emerging themes, accomplished through collaborative and frequent discussions between the researchers. This iterative dialogue was crucial for verifying the accuracy and relevance of the initial codes, which were then methodically organized into coherent patterns. Third, the well-defined themes underwent critical examination and were named, culminating in the integration of these themes into a comprehensive narrative. This narrative adeptly captured the depth and complexity of the participants’ experiences, illustrating the nuanced findings of our study. Facilitated by Nvivo software, this approach ensured the systematic organization and exploration of the data, thereby underscoring our commitment to methodological rigor throughout the research process.

## 5 Results

### 5.1 RQ1: How do the mutual friend rules on Moments affect users?

#### 5.1.1 Response visibility and friendship jealousy

Our participants felt that the limited response visibility protected users’ privacy to some extent and facilitated interactions between mutual friends. However, they reported that it also highlighted relationship comparisons between users, leading to friendship jealousy. Our participants said that the strength of their jealous feelings varied according to the strength of the relationship.

##### 5.1.1.1 Feeling jealous as a poster

Many of the participants said that they compared their posts with those of others in terms of the responses from mutual friends. When they received fewer responses, they felt jealous, unhappy, and disappointed, most likely because their self-esteem was threatened (White, 1981). For example, P13 said, “When I share a post and one of my friends doesn’t give me a like, but I see that she’s responded to another friend’s post, that makes me unhappy.” Previous research has also found that individuals who experience rejection or a need for popularity are more likely to feel jealous (Kim et al., 2017; Murphy and Russell, 2018). Our participants said that they wondered why they had not received a response and suspected that the content they had posted was less interesting than that of other posters. They were also prone to re-evaluating their relationships with those mutual friends, perhaps thinking that other posters had stronger relationships with those friends. P16 said, “I wondered why they didn’t like my post, whether the content I posted wasn’t interesting. I also thought they must have better relationships than me.” Previous research has suggested that jealous individuals often feel that they have failed compared to their “rival” (Guerrero et al., 1995). Our participants also said that they were more likely to feel jealousy in close relationships than in more distant ones. That is, they did not care much about the responses of mutual friends with whom they did not have close relationships. Previous studies have also indicated that jealous feelings are more likely to arise in highly valued relationships.

##### 5.1.1.2 Feeling jealousy as an audience member

Many of our participants said that they habitually compared the responses of other commenters who were mutual friends. For example, they said that if a poster responded to the comments of other mutual friends but not to theirs, they felt angry with the poster and jealous of the other commenters. For instance, P13 said, “When facing this situation, I feel very angry and may even delete the relevant comment. Then the next time I see the poster offline I ask them why didn’t reply to my comment.” This is consistent with prior research suggesting that when individuals feel jealous in the context of a friendship, they experience a compound of emotions that includes anger and sadness (Krems et al., 2021; Parker et al., 2010).

The results regarding relationship strength were not consistent. Some of our participants said that they cared more about whether close friends replied to them because the opinions of good friends were most important. For instance, P16 said, “If we are good friends, I wonder why they didn’t reply. Did I do something wrong?” This is consistent with previous research that has shown that individuals experience jealousy when a valued friend’s involvement with someone or something does not include them (Aune and Comstock, 1991). Other participants said that they focused more on the replies of people with which they had more distant relationships because they were worried about reciprocity. In social networks, reciprocity is important for relationship maintenance, especially in cases of weaker social ties (Cheikh-Ammar and Barki, 2016). These participants said that if a poster did not reply to them, they felt upset and distant from the poster. P17 said, “If the relationship is weak, and I comment on your post but you don’t reply to me, that’s very rude.” These participants were more tolerant of non-responses from good friends. Our participants were also concerned that their response behaviors would lead posters to make similar comparisons. To avoid this, some would make sure that they responded to all of the posts of mutual friends.

##### 5.1.1.3 Feeling under surveillance as an audience member

If an audience member responds to a poster with whom he or she has many mutual friends, the audience member may feel that he or she is under surveillance by these mutual friends. On the one hand, some participants said that they were concerned that their comments would be judged by certain mutual friends, especially work supervisors. P21 said, “We all added our supervisors on WeChat, so we need to be careful when commenting on others’ posts.” P6 said that he carefully considered whether his comments would be appropriate because he knew they would be viewed by mutual friends. Other participants were more worried about their comments being viewed by people who turned out unexpectedly to be mutual friends. P7 said, “I’m definitely a little cautious. Because people I who I think don’t know each other might actually be good friends.” This means that mutual friend rules may lead users to interact with each other more cautiously.

#### 5.1.2 Response notifications and annoyance

To encourage interactions among users, Moments developed a response notification function that notifies users when other users have interacted with the same post. However, many of the participants said that they did not like this function because they found the notifications irritating. P15 said, “It’s a little annoying. It pops up as a lot of red dots for me, some of which are for likes and comments on my posts and some of which are for things that have nothing to do with me. I click on them, but they turn out to be messages related to other people.” Some of our participants said that they preferred to receive notifications for comments but not likes. This response notification feature also seemed to aggravate relationship comparisons between mutual friends.

Other participants considered this function useful, as it enabled them to see how other mutual friends interacted with a certain poster in real time. P9 said, “It tells me about others’ responses to him. I think it’s good.” Other participants said that the notification function helped them identify mutual friends. P8 said, “Sometimes [through this function] I suddenly find out that two people I know are friends.” Users may also attempt to make guesses about someone’s personality or hobbies based on mutual friends. P2 said, “I find that I can speculate about a person I don’t know based on our mutual friends, as there are often common characteristics among mutual friends.” This is consistent with prior research suggesting that people’s friends may partially reflect their identity (Donath and Boyd, 2004; Green et al., 2018). Generally, the participants who had a large number of WeChat friends considered this function more useful.

#### 5.1.3 Information dissemination and embarrassment

Many SNS users engage in impression management through audience segmentation (e.g., using their friend list on Facebook) to direct certain information to certain audiences (Vitak et al., 2015; Zhang et al., 2021a). However, someone who has been blocked by a poster may still be able to find out information from a mutual friend who has access to the poster’s content (a situation shown in Figure 3). This may result in negative feelings and even conflicts. For instance, P17 said, “I sometimes feel guilty about segmenting the audience, as I don’t want to tell someone something directly but I know they’ll find out later. For example, I recently blocked my parents when I posted about my romantic relationship. But the next week when I went home, my father asked me whether I was in a relationship. I don’t know who told him, but I know it must have been someone who saw my post and then told my father.” This is consistent with previous research that has found that information dissemination between mutual friends is common in situations in which one friend has been blocked (Li et al., 2018). Posters may then feel bad when the person they have blocked finds out. P20 said, “Once I had a conflict with my roommates, so I blocked them when I shared a post. However, one of my friends told them about my post, so they found out that I had blocked them, which made me feel very embarrassed.” As Moments does not directly give users information about mutual friends, they can only find out through online interaction cues or offline information. This means that the information provided by Moments about mutual friends may increase the difficulty and uncertainty in users’ audience management strategies.

### 5.2 RQ2: How do users deal with mutual friend issues on Moments?

A summary of the mutual friend issues that Moments users face as both a poster and an audience member, along with their strategies to solve these issues, is shown in Table 3.

**TABLE 3:** Mutual friend issues and users’ coping strategies.

User role	Mutual friend issue	Strategies	Relevant rules
Poster	• Feeling jealous when they receive fewer responses from mutual friends	• Delete certain posts • Disclose less information	• Response • visibility
	• Feeling embarrassed when blocked users find out information from mutual friends	• Strictly manage audience • Disclose less information • Limit the display duration of posts	• Information • dissemination
Audience	• Feeling jealous when a poster replies only to other mutual friends	• Delete certain comments • Reduce interactions with the poster	• Response • visibility
	• Feeling under surveillance when making a comment	• Leave a comment cautiously	
	• Feeling annoyed when receiving notifications about one-click responses to posts	• Reduce responses to people with many mutual friends • Undo the response (cancel the like or delete the comment)	• Response • notification

#### 5.2.1 Posters’ coping strategies

The mutual friend issues faced by posters are mostly caused by limited response visibility and unwanted dissemination of information, the effects of which may make them feel jealous or embarrassed.

Compared to public response visibility, response visibility based on mutual friends appears to increase the intensity of relationship comparisons among users. When our participants posted content, they reported that they often felt jealous when their posts received fewer responses than those of mutual friends. To deal with these negative experiences, they often deleted certain posts or decided to disclose less information in the future. P17 said, “If I and A share a post at the same time, I compare the number of likes and comments from mutual friends on my post to the number on A’s post. I feel humiliated if I receive fewer responses than A, and I often delete the post later on.” P19 also said that she would reduce the frequency of her posts on Moments if she received fewer responses than other posters. This may be because social rejection threatens individuals’ self-esteem, potentially resulting in defensive reactions (Schröder-Abé et al., 2007).

To solve the problem of unwanted information dissemination between mutual friends, posters draw on the following three strategies. First, they may strictly manage their audience. That is, if there is one person they want to block, they block all of the friends in that circle. For instance, P8 said, “When I segment the audience, I block the whole group.” However, there are often pairs of mutual friends that users do not know about, which means that this strategy is not completely reliable. Second, they may reduce the amount of information they disclose on Moments. P18 said, “If I’m thinking about sharing a post but would need to block someone to share it, this doesn’t feel safe to me, so I end up not disclosing the information at all.” Finally, because even strict audience management cannot completely prevent unwanted information dissemination and engaging in audience segmentation can be cumbersome and time-consuming (Vitak and Kim, 2014), some users may calibrate their privacy settings to limit the display duration of their posts (e.g., the content might be visible for only 3 days). This strategy echoes the findings of Zhang et al. (2021b) and Huang et al. (2020). By doing this, users can at least partially hide the evidence of their blocking behaviors. For instance, P9 said, “I don’t block people because I’m worried I might be found out. So I set my posts to be visible for only 3 days.” None of these strategies is completely satisfactory, which means that Moments users currently lack a reliable way to avoid unwanted information dissemination between mutual friends.

#### 5.2.2 Audience members’ coping strategies

Moments users often complain about the mutual friend issues they experience as audience members. The response visibility based on mutual friends feature, for example, causes them to make relationship comparisons based on the poster’s responses. Our participants said that they felt jealous when a poster replied to another mutual friend but not to them. These negative experiences frequently led them to delete certain comments or reduce the frequency of their subsequent interactions with the poster. P20 said, “[In that situation], I conclude that the poster forgot about the comment or didn’t see it and I secretly delete my comment.” P3 indicated that if this occurred she would stop leaving comments for that poster in the future. This is consistent with previous research that has found that jealousy reduces connectedness, positive interactions, and prosocial behavior in relation to friends (Deutz et al., 2015). The mutual friend rules also appear to make users feel that they are under surveillance, which leads them to take increased care with their comments or polish the content that they post. P18 said, “Due to the rules related to mutual friends, I’m very cautious when I comment on others’ posts, and I may not really express what I want to express.” Some of the participants reported using this rule for impression management, deliberately choosing to direct selected information toward mutual friends. P7, for example, said, “I often deliberately say something that I know our mutual friends would be interested in.”

The response notification feature can cause audience members to feel annoyed when they receive notifications about the responses to other people left by mutual friends. To deal with this, some of the participants said that they might only respond to people with whom they share many mutual friends or they might decide not to respond to the post at all. P3 explained, “A post with photos receives many likes from others, so I really don’t want to like the post. If I do, I know I’ll receive heaps of notifications about others’ responses to this post. So I choose to scroll past it.” P13 also said that she withdrew her likes on posts that were receiving many likes from others. Most of the participants said that they only wanted to receive notifications about others’ comments or about things related to the participants themselves. These reflections suggest that users are uncomfortable with excessive levels of interaction with mutual friends and do not want to be pushed by the platform to interact with others.

## 6 Discussion

We investigated how mutual friend rules on Moments affect users and how users deal with mutual friend issues. We found that limited response visibility highlights information about the interactions between mutual friends, which may cause friendship jealousy and reduce user engagement (e.g., users may disclose less information or reduce the frequency of their interactions with others). Response notifications regarding the activity of mutual friends may annoy users and lead them to leave fewer responses. These two mutual friend rules are associated with interactional needs, and our findings reflect the fact that users have private and variable interactional needs. The mutual friend rules on Moments may also lead to the unwanted dissemination of information, potentially resulting in embarrassment or conflicts. To avoid this possibility, users often strictly manage the audiences of their posts, disclose less information, or limit the display duration of their posts. This suggests that platforms may need to reconsider whether they offer such features.

### 6.1 Mutual friend interaction needs

#### 6.1.1 Needs for private interaction

The visibility affordance of social media enables users to view others’ posts and activity information easily (Treem and Leonardi, 2013). According to visual rhetoric theory, visual communication traces (e.g., likes and comments) have various interpretations, which in turn have both conscious and unconscious effects on users’ attitudes toward and behaviors on social media (Cheikh-Ammar and Barki, 2016; Cyr et al., 2009). For example, the “like” button signals relational investment and support (Trieu and Baym, 2020).

However, due to the existence of certain features related to mutual friends, some interaction information may be misinterpreted and cause friendship jealousy, resulting in negative outcomes (e.g., disconnectedness and conflicts) (Deutz et al., 2015). This is more common on social networks than in conventional friendships, where interactions occur in private settings with less public visibility (Parks and Floyd, 1996). Conventional friendships often involve deeper emotional exchanges and trust-building, which reduces the likelihood of misunderstandings and jealousy (Fehr, 2004). In contrast, the openness of social networks can amplify subtle relational dynamics. A simple “like” or comment, routine in conventional friendships, can be misinterpreted in a public social network setting, causing jealousy or conflicts (Marshall et al., 2013; Rydell et al., 2004). This underscores the need for greater emphasis on privacy in social network interactions, especially when mutual friends are involved.

As such, some of our participants reported that they wished to respond with private likes or comments to the posts of people with whom they shared many mutual friends. This may be because individuals’ interaction rules in relationships vary according to the strength of the relationship (Bryant and Marmo, 2012). By posting private responses, users can achieve their relationship maintenance goals and partially reduce interpersonal uncertainty. Trieu and Baym (2020) discussed this kind of private feedback in relation to Snapchat, suggesting that it may reduce feedback-related expectations and pressure related to self-presentation; however, their discussion did not include a consideration of users’ privacy needs in relation to mutual friend features. We highlight the way that mutual friend issues give rise to user needs for privacy in their interactions on social media.

#### 6.1.2 Needs for relevant interaction

Most SNSs feature response notifications that aim to facilitate the interactions between users based on information about mutual friends. Although the connection affordance of social media may aid in establishing and maintaining relationships (Treem and Leonardi, 2013), it may also lead to interpersonal stress (Fox and Moreland, 2015). That is, users may be pushed by the platform to connect with others in situations in which they do not wish to connect. In conventional friendships, interactions are typically based on mutual consent and trust, free from external pressures (Duck, 1994). These interactions are often spontaneous and driven by emotional support, without the influence of technology or platforms (Rawlins, 1992). Unlike social media, where notifications can compel unwanted interactions, traditional face-to-face connections occur naturally and without such disruptions (Parks and Floyd, 1996; Valkenburg and Peter, 2011).

Our participants complained that the Moments response notifications based on mutual friends were particularly annoying. Most of the participants disliked this function and said that it bothered them to receive constant notifications about mutual friends’ activity on the platform. When our participants interacted with a post, they preferred to receive notifications for comments but not for one-click reactions, such as likes. It has previously been argued that comments are associated with focused interaction and can therefore provide valuable information, whereas likes are a type of unfocused interaction and carry no specific information (Hall, 2018), an insight that may explain this result. Some of our participants said that they only wanted to receive information about interactions that were relevant to them. This is consistent with previous research that has found that users want to choose when and how they interact with others on social media and do not want to feel obligated to interact (Bryant and Marmo, 2012; Fox and Moreland, 2015). Interaction with friends on social media is still important for obtaining social support and maintaining relationships (Cheikh-Ammar and Barki, 2016). Platforms should therefore offer mutual friend features that satisfy these needs.

### 6.2 Mutual friend transparency

Audience transparency refers to “the extent to which a platform affords user awareness of who is actually in the audience for persona-linked content” (DeVito et al., 2017). Although researchers have suggested that audience transparency in social media has benefits, they have mainly focused on resolving the disparity between users’ imagined audiences and their actual audiences through audience visualization (DeVito et al., 2017; De Wolf et al., 2015). Mutual friend rules in the context of high audience transparency have not previously been the focus of attention (Li et al., 2018). We extend the literature on audience transparency by highlighting transparency related to mutual friends. Mutual friend transparency affects the speed and pathways of information dissemination on social media. Higher transparency helps users better manage who can see their content, reducing the risk of unintended information sharing (Treem and Leonardi, 2013; Ellison et al., 2011). Low transparency can lead to privacy breaches and social conflicts (Vitak, 2012). Therefore, enhancing transparency enables more precise control over information dissemination, improving user trust and satisfaction (Boyd, 2010).

However, most social media platforms do not give users details about mutual friends. Many simply provide a mutual friend count (i.e., a statement of the number of friends the two users have in common) when a user adds a new friend. Nonetheless, the existence of mutual social media connections between users can affect their audience management strategies and result in unintended information dissemination (Li et al., 2018; Zhang et al., 2023). Our participants were worried about information dissemination between mutual friends, so they often segmented their audience by separating mutual friends into different circles. However, this segmentation is difficult to achieve when a user is unaware of all of the connections they share with another user. Some of our participants reported that they were careful about the digital traces they left on social media due to the possibility of shared connections they did not know about. If more information about mutual friends is available to users when they add a new friend, they can segment their audience more strictly to avoid the unintended dissemination of information. We follow Li et al. (2018) in proposing that platforms provide a list of mutual friends when users are adding a new friend to help them better manage their audience.

### 6.3 Design recommendations for dealing with mutual friend issues

Based on our results, we recommend that social media platforms integrate features and rules that help users deal with mutual friend issues. First, providers should give users more freedom to choose how they interact with mutual friends. Our findings indicate that users want the option to interact privately with posts so they can avoid creating negative experiences for mutual friends. To address this desire, platforms could enable users to set the visibility of feedback, making it either private or public. Our results also suggest that users only want to receive notifications that are directly relevant to them. They are annoyed when they receive notifications about mutual friends liking a post they have already interacted with. Some of the participants said that they preferred to receive notifications about comments but not likes. Accordingly, platforms could allow users to choose which types of notifications they would like to receive (e.g., all interaction information, comments only, or no notifications at all).

Our findings also highlight the important role of mutual friend transparency in users’ audience management strategies. To increase mutual friend transparency, providers could provide lists of mutual friends or mutual friend visualizations when users are adding a new friend. Doing this would allow users to better segment their audience and thereby avoid the unintended dissemination of information between mutual friends. However, some of our participants reported that they wanted to know which connections they shared with a new friend but did not want the new friend to have the same information about them. They considered information about their friends to be private, perhaps because one’s friends may carry information about personal identity (Donath and Boyd, 2004; Green et al., 2018). To resolve this dilemma, we can follow the “equal rights principle”: that is, if users want to know others’ mutual friend information, they must allow others access to the same information about them. For example, on TikTok, if users want to know who visits their profiles, their activity can also be viewed by others. If they cancel this function, their activity is private but they cannot see who has visited their profile.

## 7 Limitations and future directions

This study has several limitations. We only examined the three mutual friend rules on WeChat Moments, which may limit the generalizability of our findings. Although, similar rules may exist on other social platforms, our research findings may not be entirely applicable. Additionally, certain other mutual friend rules on these platforms, such as displaying mutual friend counts, were not analyzed. We hope that future scholars will build upon our research to delve deeper into mutual friend issues on other social platforms (e.g., Facebook and Instagram). Moreover, we interviewed only 22 WeChat users, and this small sample may not represent the broader WeChat user population. Future research could investigate mutual friend issues on other social media platforms with larger sample sizes using surveys or experiments.

The results of the present study suggest several directions for future research. First, mutual friend issues on social media could be explored from other perspectives and using other methods. Our results show that features related to mutual friends may lead to negative outcomes (e.g., reducing the disclosure of information or curtailing interactions on SNSs). Previous research on the “dark side” of SNSs [e.g., Chen and Lee (2013), Fox and Moreland (2015)], coupled with our results, opens avenues for further research. Second, future research could focus on friendship jealousy in social media, as we show that users may experience and express jealousy related to their online friends. Although previous studies (Demirtaş-Madran, 2018; Hudson et al., 2015; Orosz et al., 2015) have shown that the use of SNSs can evoke feelings of jealousy, they mainly focused on romantic relationships. Almost no attention has been paid to friendship jealousy caused by SNSs. Our findings suggest some initial directions, but these could be extended further. Finally, it would also be interesting to explore the roles of mutual friend interactions and mutual friend transparency in social media. An exploration of these phenomena could yield useful guidelines for platforms when they design specific settings or algorithms to satisfy users’ needs. As many users benefit from the ephemerality affordance in social media to support their self-presentations and interaction needs (Luria and Foulds, 2021; Zhang et al., 2022), future studies could also investigate how the ephemerality affordance affects mutual friend interactions.

## 8 Conclusion

As the audience size of SNS users increases, features related to mutual friends could increase the difficulties that users encounter in their self-presentation and interactions on SNSs. Few studies to date have examined the mutual friend issues that arise on social media. We explored how various mutual friend rules affect users of WeChat Moments and how users deal with these mutual friend issues. Our findings show that the existing mutual friend rules on Moments (response visibility, response notifications, and information dissemination) may cause users to feel jealous, annoyed, or embarrassed. We found that to resolve these issues, users often reduce the amount of information they disclose or the frequency of their interactions with others on the platform. Based on these findings, we proposed several design considerations to help platforms better satisfy users’ interaction needs.

## Data Availability

The raw data supporting the conclusions of this article will be made available by the authors, without undue reservation.

## Appendix

**APPENDIX A:** General interview questions.

Parts	Questions
General use of Moments	• Do you frequently use WeChat Moments? • Is your primary focus on publishing content or browsing through others’ posts? • How many friends do you have on WeChat, and what types of relationships do they represent?
How mutual friend rules impact users	• Have you ever discovered through WeChat Moments that two of your friends are also connected? If so, how did you feel when you realized this? • Have you ever encountered an awkward situation after posting on social media because you were unaware that two of your friends were mutual connections? If so, could you describe what happened? • How do you handle the possibility of a friend sharing your post with a mutual connection that you have blocked? • Do you believe that having mutual friends influences how you post on social media? • Have you ever used the 3-day visibility feature on your Moments? Do you think limiting visibility to a few days effectively prevents others from discovering they’ve been blocked? • Do you usually care about the number of likes and comments you receive when posting on WeChat Moments? If the interaction is low, would you feel unhappy or embarrassed? • How do you feel when you notice a mutual friend interacting with others’ posts but not with yours? Would this affect your future interactions with them? • When you like or comment on a friend’s post, and mutual friends also interact with the same post, causing a small red dot to appear on your Moments main interface, do you find this feature useful? Why or why not? • Do you think the presence of mutual friends affects your behavior when it comes to liking and commenting on posts? • How do you feel when you see interactions between your mutual friends on your WeChat Moments?
Suggestions for the managing mutual friends	• What are your thoughts on the idea of a feature in WeChat that would notify you of mutual friends whenever you add someone new? • Do you have any suggestions for managing mutual friends on WeChat? • Are there any features or mechanisms you wish WeChat would introduce?
